# Inflammatory markers, the tryptophan-kynurenine pathway, and vitamin B status after bariatric surgery

**DOI:** 10.1371/journal.pone.0192169

**Published:** 2018-02-05

**Authors:** Monika H. E. Christensen, Dag J. Fadnes, Therese H. Røst, Eva R. Pedersen, John R. Andersen, Villy Våge, Arve Ulvik, Øivind Midttun, Per M. Ueland, Ottar K. Nygård, Gunnar Mellgren

**Affiliations:** 1 Department of Clinical Science, University of Bergen, Bergen, Norway; 2 Department of Medicine, Haukeland University Hospital, Bergen, Norway; 3 Medical Department, Førde Hospital Trust, Førde, Norway; 4 Department of Heart Disease, Haukeland University Hospital, Bergen, Norway; 5 Sogn og Fjordane University College, Førde, Norway; 6 Center of Health Research, Førde Hospital Trust, Førde, Norway; 7 Department of Surgery, Voss Hospital, Bergen Health Trust, Voss, Norway; 8 Bevital AS, Bergen, Norway; 9 Laboratory of Clinical Biochemistry, Haukeland University Hospital, Bergen, Norway; 10 KG Jebsen Center for Diabetes Research, Department of Clinical Science, University of Bergen, Bergen, Norway; 11 Hormone Laboratory, Haukeland University Hospital, Bergen, Norway; Baylor College of Medicine, UNITED STATES

## Abstract

**Objective:**

Obesity is associated with increased inflammation and insulin resistance. In conditions with chronic immune activation, low plasma vitamin B6-levels are described, as well as an increased kynurenine:tryptophan-ratio (KTR). We investigated circulating tryptophan, kynurenine and its metabolites, neopterin, B-vitamins, CRP, and HbA1c in individuals with obesity before and after bariatric surgery.

**Methods:**

This longitudinal study included 37 patients with severe obesity, scheduled for bariatric surgery. Blood samples were taken at inclusion and at three months and one year postoperatively.

**Results:**

We observed significant positive correlations between HbA1c and both 3-hydroxy-kynurenine and 3-hydroxyanthranilic acid at inclusion. After surgery, fasting glucose, HbA1C and triglycerides decreased, whereas HDL-cholesterol increased. Tryptophan, kynurenine and its metabolites, except for anthranilic acid, decreased during weight loss. The KTR and CRP decreased while vitamin B6 increased during the year following operation, indicating reduced inflammation (all p<0.05).

**Conclusions:**

In patients with obesity subjected to bariatric surgery, levels of 3-hydroxykynurenine and 3-hydroxyanthranilic acid seemed to be positively correlated to impaired glucose tolerance. One year following surgery, plasma levels of the kynurenine metabolites were substantially decreased, along with a metabolic improvement. The relation of circulating kynurenine pathway metabolites with biomarkers of metabolic impairment in patients with obesity needs further evaluation.

## Introduction

Severe obesity is associated with highly elevated risk of adverse health outcomes [1] including type 2 diabetes (T2D), hypertension, dyslipidaemia, and cardiovascular diseases, as well as premature death and several forms of cancers [2]. Weight loss reduces existing co-morbidities in patients with obesity and may cause remission of T2D [3]. Compared to lifestyle intervention and pharmacological treatment, bariatric surgery leads to greater weight loss and metabolic improvement in patients with severe obesity [4].

Adipose tissue has important endocrine functions regulating energy homeostasis, insulin sensitivity, lipid and carbohydrate metabolism [5, 6]. In obesity, an increased number of pro-inflammatory immune response cells are found in adipose tissue. Consequently, increased levels of pro-inflammatory cytokines are found [7–9]. Inflammation in adipose tissue seems to promote and accentuate insulin resistance, an early feature in the progression from normoglycemia to overt T2D [10].

**Fig 1:**
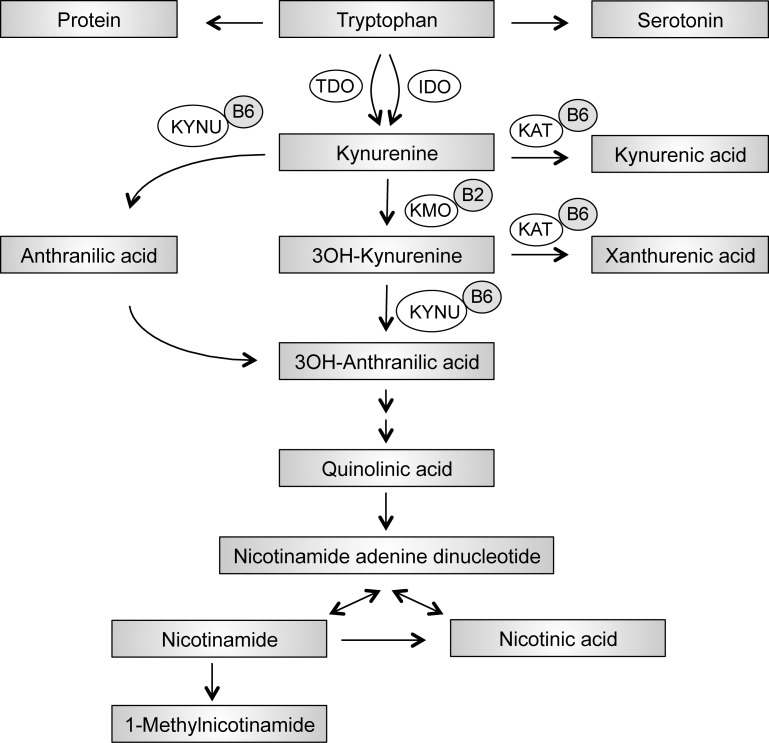
The kynurenine pathway of tryptophan metabolism. IDO, indoleamine 2,3-dioxygenase; TDO, tryptophan 2,3-dioxygenase; KAT, kynurenine aminotransaminase; KMO, kynurenine 3-monooxygenase; KYNU, kynureninase; 3OH-kynurenine, 3-hydroxy kynurenine; 3OH-anthranilic acid, 3-hydroxy anthranilic acid; B6, vitamin B6 (pyridoxal 5`-phosphate); B2, vitamin B2 (flavin adenine dinucleotide).

The essential amino acid tryptophan is obtained from the diet. Tryptophan is mainly metabolised through the kynurenine pathway and is a source for NAD^+^, an essential cofactor in energy metabolism. A smaller portion of tryptophan is used to synthesize the neurotransmitters serotonin and melatonin (Fig 1)[11]. In the liver tryptophan is metabolized by the enzyme tryptophan 2,3 dioxygenase (TDO), induced by stress and steroids. Plasma levels of kynurenine, however, might be more closely linked to the activity of the inflammation-induced enzyme indoleamine 2,3-dioxygenase (IDO) [12]. IDO is ubiquitously expressed and induced by inflammatory cytokines such as IFNγ [13]. The ratio between circulating kynurenine and tryptophan (KTR) in plasma is thus often used as a measure of IFN-γ activation. Neopterin, which is released in large quantities by activated macrophages, is another well-established marker of IFN-γ activity [14, 15]. Kynurenine metabolites have distinct roles in immune-regulation or can be neuroactive [16, 17]. Several key enzymes in the tryptophan-kynurenine metabolic pathway require pyridoxal 5'-phosphate (PLP, vitamin B6) or flavin adenine dinocleotide (FAD, vitamin B2) as cofactors (Fig 1). PLP is the most commonly used serum marker of vitamin B6 status and reflects the body store of this vitamin [18]. In conditions associated with chronic inflammation low plasma levels of PLP have been described [19].

Obesity leads to low-grade chronic inflammation and is associated with increased risk of metabolic diseases. Inflammatory markers may be useful as a diagnostic tool to identify subjects with obesity at high risk of developing metabolic diseases. In this study we present a tryptophan-kynurenine metabolite profile in plasma samples of patients with morbid obesity at baseline, 3 and 12 months after bariatric surgery.

## Materials and methods

### Participants

The study includes 37 subjects undergoing bariatric surgery, either laparoscopic sleeve gastrectomy (LSG) or biliopancreatic diversion with duodenal switch (BPD-DS), at the Department of Surgery at Førde Hospital, Norway. A total of 40 subjects were recruited to the study, but three patients withdraw from surgery. LSG was performed by resecting along a size 32 French tube from the pylorus to the cardia creating a gastric volume of 100 ml or less. BPD-DS was performed by creating a slightly greater gastric tube, excluding 60% of the small bowel and making a common channel of 10% of the small bowel length. Further details on the surgical procedures can be found in previous publications [20, 21]. Criteria to be scheduled for bariatric surgery were age 18–60 years, BMI ≥ 40 kg/m^2^ or ≥ 35.0 kg/m^2^ if at least one obesity-related comorbidity (such as T2D, hypertension or dyslipidaemia) was present. T2D was diagnosed if fasting plasma glucose was ≥ 7.0 mmol/L and/or plasma glucose measured 2 hours after intake of 75g of glucose was ≥ 11.1 mmol/L. Eligiblity criteria also included no alcohol or drug abuse and no major psychiatric disorder. Participants were recruited during the period from September 2010 to May 2012. In general, patients who had T2D treated with insulin, were using two or more antihypertensive drugs and/or received lipid-lowering treatment were scheduled for BPD-DS. Inclusion and exclusion criteria for taking part in the study were identical to the indications for bariatric surgery. None of the patients dropped out of the study. Thus, any missing data is due to patients not being able to attend follow-up appointments for personal reasons or random errors in the data collection. All patients were alive at the end of the study.

Clinical examination and blood sampling were performed two days before surgery and repeated after 3 and 12 months. Weight was measured to the nearest 0.1 kilograms on electronic scales with participants dressed in light clothing, and height in standing position without shoes was measured to the nearest millimetre. Body mass index (BMI) was calculated as weight divided by the square of body length (kg/m^2^). Percentage excess body mass index loss (%EBMIL) from baseline to the one-year follow-up was calculated using the formula 100-((Follow-up BMI-25/Beginning BMI-25)x100).

At inclusion 10 out of 37 patients used multivitamin supplements. Following surgery, all patients were instructed to take one multivitamin supplement daily. The subset undergoing BPD-DS were also instructed to take 30.000 IE of 25(OH)D and 2 gram calcium carbonate per day. At 3 months after surgery 28 patients reported the use of multivitamin supplements, containing a daily dose of 1.6–1.7 mg of vitamin B2 (riboflavin), 18–20 mg vitamin B3 (niacin), and 1.2–2.0 mg vitamin B6 (pyridoxine), in addition to other vitamins and minerals. After 12 months 32 patients used supplements.

The study was approved by the Western Norway Regional Committee for Medical Research Ethics (ref.nr. 2009/2174). All enrolled subjects provided informed written consent, and the study was performed according to the principles of the Declaration of Helsinki.

### Biochemical analyses

All blood samples were drawn after a fasting period of at least twelve hours. Glucose, HbA1c, CRP, total cholesterol, HDL-cholesterol, and triglycerides were measured immediately at the Laboratory of Clinical Chemistry at Førde Hospital, using the Architect system from Abbot Diagnostics (North Chicago, Illinois, USA). Samples stored in the biobank were frozen within 60 minutes and kept at -20°C for 1–2 days before being stored at −80°C.

Plasma concentrations of tryptophan, kynurenine, kynurenic acid, anthranilic acid, 3-hydroxykynurenine, xanthurenic acid, 3-hydroxyanthranilic acid, quinolinic acid, neopterin, pyridoxal 5´-phosphate (PLP), riboflavin, nicotinamide N^1^-methylnicotinamide, and creatinine were analysed by liquid chromatography/tandem mass spectrometry (LC-MS/MS) [22] at Bevital A/S, Bergen, Norway (www.bevital.no) by laboratory personnel blinded to the clinical characteristics and identity of the patients. Estimated glomerular filtration rate (eGFR) was calculated according to the Modification of Diet in Renal Disease (MDRD) Study Group formula [23].

### Statistical analysis

Continuous variables are reported as median (25th-75th percentiles) and categorical variables as numbers (percentages). Where distributional assumptions were violated, measures were either log10 transformed (kynurenic acid, anthranilic acid, 3-hydroxykynurenic acid, xanthurenic acid, 3-hydroxyanthranilic acid, quinolinic acid, pyridoxal 5`-phosphate, riboflavin and N^1^-methylnicotinamide) or ranked (CRP) before used in parametrical tests. To assess changes in baseline characteristics between inclusion and one year after surgery Friedman´s test was used. Changes over time between repeated end-point measures were assessed with a random intercept mixed model, adjusted for operation method and multivitamin supplement use. Correlations among continuous variables were assessed by Spearman rank correlation, corrected for age, gender and eGFR, as well as operation method when analysing correlations between changes over time. All tests were two-sided and p-values < 0.05 were considered to be statistically significant. Statistical analyses were performed using SPSS statistics 22 for Mac (IBM Corporation, New York, NY, USA).

## Results

### Baseline characteristics

Median age (25^th^-75^th^ percentile) of the 37 participants was 48 (42.5–53.5) years, 25 (68%) participants were females, and median BMI (25^th^ -75^th^ percentile) was 43.8 (40.9–47.6) kg/m^2^. At inclusion 38% had diabetes, of which one patient had type 1 diabetes mellitus, 17% hypertension and 12% hypercholesterolemia. LSG was performed on 25 patients (68%) whereas 12 underwent BPD-DS (Table 1). For two of the participants EDTA-plasma samples at baseline were missing, though there were blood samples of these patients at three months and one year after surgery.

**Table 1:** Baseline characteristics of the study population according to bariatric surgical procedures.

	All patients		LSG		BPD-DS		
	(*n* = 37)		(*n* = 25)		(*n* = 12)		p-value
Females (%)*	25	(68%)	18	(72%)	7	**(**58%)	0.41
Age (years)**	48.0	(42.5–53.5)	44.0	(39.5–51.5)	52.5	(48.3–58.5)	0.011
BMI (kg/m^2^)**	43.8	(40.9–47.6)	45.4	(41.5–47.6)	41.9	(38.3–51.0)	0.98
Smoking (%)*	14	(38%)	9	(36%)	5	(41%)	0.74
Fasting glucose (mmol/L)	5.80	(5.30–8.63)	5.50	(4.95–6.30)	8.40	(5.93–15.6)	0.066
HbA1C (%)	5.60	(5.50–6.90)	5.60	(5.30–6.10)	7.15	(5.68–10.4)	0.003
Cholesterol (mmol/L)	4.90	(4.00–5.55)	4.75	(3.93–5.83)	4.90	(4.20–5.25)	0.684
Triglycerides (mmol/L)	1.44	(1.25–1.77)	1.43	(1.24–1.65)	1.49	(1.22–2.21)	0.286
HDL-cholesterol (mmol/L)	1.06	(1.00–1.20)	1.10	(1.00–1.28)	1.00	(0.90–1.20)	0.101
Creatinine (umol/L)	68.6	(59.1–76.0)	68.8	(59.2–74.7)	64.1	(58.5–82.7)	0.506
eGFR (mL min^-1^per 1.73m^2^)	94.3	(80.1–106)	94.2	(80.8–106)	97.1	(73.0–108)	0.140
Diabetes*	13	(38%)	6	(24%)	7	(67%)	0.041
Diabetes medication*	10	(30%)	3	(12%)	7	(67%)	0.003
Hypertension*	17	(46%)	11	(44%)	6	(50%)	0.73
Hypercholesterolemia*	12	(32%)	6	(24%)	6	(50%)	0.11

Values are given as median (25^th^ to 75^th^ percentile), or numbers (percentages). LSG, laparoscopic sleeve gastrectomy; BPD-DS, biliopancreatic diversion with duodenal switch; BMI, body-mass index; eGFR, estimated glomerular filtration rate; Diabetes medication, if patents used oral antidiabetic drugs or insulin; Hypertension, if using one or more antihypertensive drugs; Hypercholesterolemia, using lipid lowering medication. p-values are based on Anova, adjusted for age, gender and BMI.

*p-values are based on chi square test

**p-values are based on students t-test

Patients selected for BPD-DS were older than the LSG patients and more frequently used anti-diabetic drugs. This subset also had higher levels of HbA1c, tryptophan and 3-hydroxyanthranilic acid. No other statistically significant differences in baseline characteristics according to bariatric surgical procedures were observed (Table 2).

**Table 2:** Circulating levels of kynurenines, neopterin and B vitamins at inclusion of patients undergoing bariatric surgery.

		All		LSG		BPD-DS		
		(*n* = 35)		(*n* = 23)				(*n* = 12)		p-value
**Tryptophan and kynurenines**								
	Tryptophan (μmol/L)	65.0	(60.1–73.4)	64.4	(59.9–72.5)	71.6	(64.4–78.3)	0.029
	Kynurenine (μmol L)	1.68	(1.38–1.91)	1.58	(1.35–1.95)	1.77	(1.52–1.88)	0.483
	Kynurenic acid (nmol/L)	58.7	(49.3–71.0)	55.3	(47.5–67.0)	71.0	(53.6–78.7)	0.245
	Anthranilic acid (nmol/L)	16.7	(13.5–20.0)	14.7	(13.3–17.9)	19.6	(16.7–25.4)	0.076
	3-Hydroxykynurenine (nmol/L)	52.0	(40.5–61.1)	50.6	(40.0–57.3)	52.1	(40.5–62.7)	0.061
	Xanthurenic acid (nmol/L)	13.3	(9.93–20.7)	12.3	(8.63–18.2)	14.6	(12.5–21.8)	0.329
	3-Hydroxyanthranilic acid (nmol/L)	38.8	(29.6–50.3)	37.2	(27.7–46.0)	50.0	(36.2–66.6)	0.042
	Quinolinic acid (nmol/L)	460	(368–578)	451	(371–508)	489	(335–640)	0.567
**Inflammatory markers**								
	KTR (nmol/μmol)	24.8	(21.7–28.9)	24.6	(21.6–30.0)	25.8	(21.7–28.9)	0.612
	Neopterin (nmol/L)	16.6	(14.6–20.1)	16.6	(14.3–18.7)	19.6	(14.6–24.4)	0.681
	CRP (mg/L)	6.00	(4.00–15.5)	6.00	(4.00–13.5)	6.00	(3.25–16.5)	0.971
**B-vitamins**								
	Pyridoxal 5’-phosphate (B6) (nmol/L)	29.9	(21.8–50.2)	37.8	(19.2–52.5)	27.8	(21.8–34.9)	0.073
	Riboflavin (B2) (nmol/L)	20.5	(14.4–31.1)	21.7	(14.8–31.7)	20.5	(12.8–29.4)	0.257
	Nicotinamide (B3) (nmol/L)	295	(211–368)	303	(221–368)	266	(180–382)	0.783
	N1-methylnicotinamide (B3) (nmol/L)	152	(104–189)	157	(104–192)	150	(101–189)	0.751

Values are given as median (25^th^ to 75^th^ percentile). LSG, laparoscopic sleeve gastrectomy; BPD-DS, biliopancreatic diversion with duodenal switch. P-values are based on Anova, adjusted for age, gender and BMI between the LSG and BPD-DS group.

### Correlations at baseline

**Fig 2:**
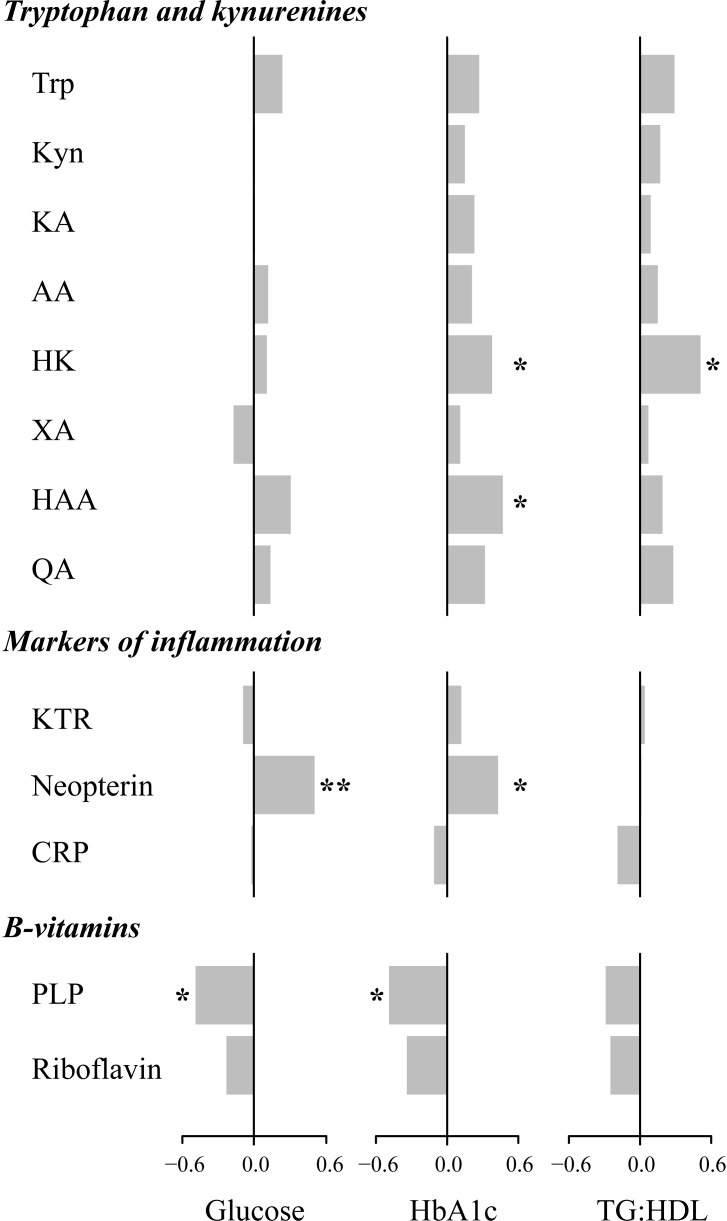
Correlations of fasting glucose, HbA1c and the triglyceride:HDL ratio with tryoptophan, kynurenine and the kynurenine metabolites, inflammatory markers, vitamin B6 and vitamin B3 at baseline. Regression coefficient based on Spearman rank correlation test, corrected for age, gender and eGFR, are shown in the figure. Tg, triglyceride; HDL, high density lipoprotein; Trp, tryptophan; Kyn, kynurenine; KA kynurenic acid; AA, anthranilic acid; HK, 3-hydroxy kynurenine; XA, xanthurenic acid; HAA, 3-hydroxy anthranilic acid; QA, quinolinic acid; KTR, kynurenine:tryptophan ratio; PLP, pyridoxal 5`-phosphate. *p-value < 0.05; **p-value < 0.005.

Correlations of plasma kynurenines, related inflammation markers and B vitamins with fasting glucose, HbA1c, TG:HDL-ratio at baseline, adjusted for age, gender and eGFR, are shown in Fig 2. None of the kynurenines were correlated to fasting glucose, whereas neopterin was strongly, positively correlated to both fasting glucose and HbA1c. Both 3-hydroxykynurenine and 3-hydroxyanthranilic acid showed strong positive associations to HbA1c, whereas 3-hydroxykynurenine was also correlated positively to the TG:HDL-ratio. PLP was negatively correlated to glucose and HbA1c. At inclusion, BMI was positively correlated to CRP (p < 0.05), but we did not find correlations to any of the other markers measured in the study.

### Changes in clinical and metabolic baseline characteristics after bariatric surgery

As expected, patients had significant weight loss after surgery. In the whole group %EBMIL was 89%. Reduction of body weight was higher in the BPD-DS group (%EBMIL 109% (85.7–114)) than in the LSG group (%EBMIL 84.7% (70.0–91.4), p<0.005). Fasting glucose (17%), HbA1c (13%), and triglycerides (39%) decreased while HDL-cholesterol (23%) increased, indicating a more favourable metabolic profile one year after surgery.

Before surgery 13 patients were diagnosed with diabetes, 10 of these used oral anti-diabetic drugs or insulin. After surgery none of the patients with T2D used insulin and only one patient used oral anti-diabetic medication (Table 3).

**Table 3:** Patient characteristics at inclusion and after bariatric surgery.

	Inclusion		12 months after surgery		
	(*n* = 37)		(*n* = 34)		p-value
Gender (% females)	25	(68%)			
Age (years)	47.1	(42.5–53.5)			
Smoking (%)	14	(38%)	10	(29%)	0.56
BMI (kg/m^2^)	43.8	(40.9–47.6)	27.1	(24.5–30.6)	< 0.001
Glucose (mmol/L)	5.80	(5.30–8.63)	4.80	(4.60–5.30)	< 0.001
HbA1C (%)	5.60	(5.50–6.90)	4.90	(4.50–5.20)	< 0.001
Cholesterol (mmol/L)	4.90	(4.00–5.55)	4.45	(3.78–5.52)	0.086
Triglycerides (mmol/L)	1.44	(1.25–1.77)	0.88	(0.70–1.07)	< 0.001
HDL-cholesterol (mmol/L)	1.06	(1.00–1.20)	1.30	(1.08–1.50)	0.002
Creatinine (umol/L)	68.6	(59.1–76.0)	63.6	(56.1–69.8)	0.003
eGFR (mL min^-1^per 1.73m^2^)	94.3	(80.1–106)	107	(85.0–123)	0.003
Diabetes	13	(38%)	2	(5%)	<0.001
Diabetes medication	10	(30%)	2	(5%)	0.002
Hypertension	17	(46%)	5	(14%)	<0.001
Hypercholesterolemia	12	(32%)	2	(5%)	0.001

Values are given as median (25^th^ to 75^th^ percentile) or numbers (percentages). BMI, body-mass index; eGFR, estimated glomerular filtration rate; diabetes medication, if patients used per oral antidiabetic drugs or insulin; hypertension, if patients used one or more antihypertensive drugs; hypercholesterolemia, using lipid lowering medication. P-values are based on Friedman´s Two.

### Kynurenine pathway metabolites and inflammatory markers after bariatric surgery

**Fig 3:**
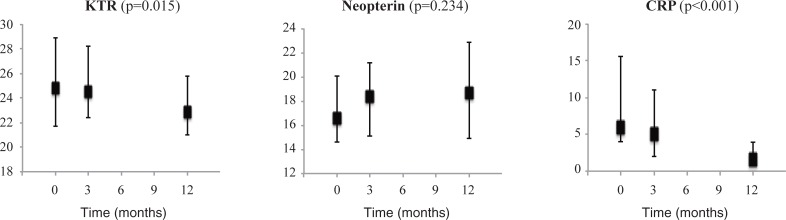
Inflammatory markers after bariatric surgery. Samples were measured at baseline, 3 months and after one year in 37 patients undergoing bariatric surgery. *P* values for trend over time are estimated with a random intercept mixed model, adjusted for type of operation and vitamin B supplementation. Data are given as median (25th to 27th percentile).

Circulating levels of metabolites in the kynurenine pathway of tryptophan metabolism at baseline, 3 and 12 months after surgery are given in Table 4. We observed decreased plasma levels of tryptophan and all the kynurenine pathway metabolites, except for anthranilic acid. These changes were generally apparent within the first three months after surgery. In contrast, anthranilic acid was unchanged at three months, but significantly increased after one year. The KTR and CRP decreased after surgery, while levels of neopterin were unchanged (Fig 3). CRP was significantly reduced already three months after surgery and continued decreasing one year post-operatively, while the KTR was unchanged at three months but reduced after one year. Analysing the LSG and BPD-DS groups separately showed the same results as in the combined group regarding CRP, neopterin, and the B vitamins measured, as well as the kynurenine metabolites, except for anthranilic acid, which was increased in the BPD-DS group but not significantly changed in the LSG group.

**Table 4:** Circulating levels of tryptophan, kynurenines and B vitamins before and after bariatric surgery.

				Time after surgery (months)				
		Inclusion		3		12		
		(*n* = 35)		(*n* = 31)		(*n* = 32)		p-value
**Tryptophan and kynurenines**								
	Tryptophan (μmol/L)	65.0	(60.1-73-4)	48.5	(41.6–54.7)	51.9	(43.8–60.3)	< 0.001
	Kynurenine (μmol L)	1.68	(1.38–1.91)	1.22	(1.06–1.33)	1.23	(1.02–1.33)	< 0.001
	Kynurenic acid (nmol/L)	58.7	(49.3–71.0)	29.2	(23.5–33.6)	30.0	(22.6–42.6)	< 0.001
	Anthranilic acid (nmol/L)	16.7	(13.5–20.0)	16.0	(13.7–22.8)	18.6	(15.2–30.7)	0.001
	3-Hydroxykynurenine (nmol/L)	52.0	(40.5–61.1)	30.7	(26.7–37.2)	30.7	(22.9–37.9)	< 0.001
	Xanthurenic acid (nmol/L)	13.3	(9.93–20.7)	3.35	(2.62–6.00)	5.09	(3.13–8.19)	< 0.001
	3-Hydroxyanthranilic acid (nmol/L)	38.8	(29.6–50.3)	15.3	(13.5–19.7)	18.4	(14.6–25.1)	< 0.001
	Quinolinic acid (nmol/L)	460	(368–578)	314	(265–368)	285	(248–356)	<0.001
**B-vitamins**								
	Pyridoxal 5’-phosphate (B6) (nmol/L)	29.9	(21.8–50.2)	33.0	(15.8–56.1)	43.4	(31.3–70.3)	0.006
	Riboflavin (B2) (nmol/L)	20.5	(14.4–31.1)	14.5	(8.84–25.7)	23.1	(16.6–26.5)	0.002
	Nicotinamide (B3) (nmol/L)	295	(211–368)	207	(155–255)	219	(166–272)	0.002
	N1-methylnicotinamide (B3) (nmol/L)	152	(104–189)	74.0	(49.7–104)	119	(81.1–219)	<0.001

Values are given as median (25^th^ to 75^th^ percentile). P-values are based on mixed models, adjusted for type of surgery and vitamin B supplementation.

Levels of PLP were not significantly changed at three months after surgery, but an increase was observed after one year. The vitamin B3 form nicotinamide as well as the catabolite N^1^-methylnicotinamide decreased three month after surgery, though the latter increased between three months and one year. Levels of N^1^-methylnicotinamide at one year post surgery did not significantly different from inclusion. Similarly, riboflavin (vitamin B2) decreased at three months, but levels one year after surgery were not significantly different from the levels at inclusion. The PLP-dependent ratios between 3-hydroxykynurenine:xanturenic acid, 3-hydroxykynurenine:3-hydroxyanthranilic acid and 3-hydroxykynurenine:kynurenic acid were all increased at three months after surgery, but decreased significantly after 12 months compared to 3 months.

In order to examine whether baseline metabolite levels could predict surgical outcomes, we analysed whether BMI, HbA1c, fasting glucose, and the TG:HDL-ratio after surgery were related to plasma kynurenines, inflammatory markers and B vitamins at baseline. Changes in BMI one year after surgery did not correlate with the metabolite levels at baseline, but we observed a positive association with changes in tryptophan (p = 0.07) and kynurenine (p = 0.014). Changes in HbA1c one year after surgery were positively correlated with baseline levels of 3-hydroxyanthranilic acid (p = 0.041) and tryptophan (p = 0.027). In addition, changes in 3-hydroxykynurenine one year after surgery were positively correlated to the changes in both HbA1c (p = 0.029) and the TG:HDL-ratio (p = 0.024). The analyses were adjusted for age, gender, eGFR, and operation method.

## Discussion

In the present study we observed a positive correlation between HbA1c and both 3-hydroxykynurenine and 3-hydroxyanthranilic acid at baseline. One year after bariatric surgery plasma levels of most kynurenine pathway metabolites were substantially decreased. In accordance with previous studies weight loss was accompanied by reduced CRP and KTR, as well as increased vitamin B6-levels. The beneficial metabolic effects one year after bariatric surgery also included decreased levels of HbA1c, triglycerides, and remission of T2D in a high proportion of patients [3, 24–26].

The role of the kynurenine pathway in obesity and during weight loss has been ambiguous. In accordance with our study, a recent report on patients undergoing either gastric banding or Roux-en-Y gastric bypass showed a decrease in tryptophan and kynurenine metabolites one year after surgery [27]. However, others have shown that in patients undergoing gastric banding levels of KTR and neopterin were unchanged two years after surgery [24]. In addition to the difference in follow-up time, a possible explanation for these contrarieties may lie in the different procedures of bariatric surgery performed. Gastric banding is shown to be a less effective method on long-term outcomes compared to LGS or BPD-DS [28], which were applied in the present study.

After LSG and BPD-DS, food intake is restricted due to reduction of the gastric volume. In patients undergoing BPD-DS the uptake of nutrients is also reduced since a part of the small bowel has been bypassed. In this study bariatric surgery was followed by a decrease in plasma tryptophan, kynurenine and all the kynurenine metabolites, except for anthranilic acid. The reduction was most prominent at three months after surgery, while levels seemed to be stable between three months and one year after surgery. Since circulating levels of tryptophan and kynurenines generally are positively related [29], the observed changes might be related to restricted food intake and decreased absorption of nutrients like tryptophan, especially during the first months after surgery. Both KTR and CRP decreased after weight loss, indicating decreased inflammation. Changes in these inflammatory markers after surgery were more prominent between three and twelve months than between baseline and three months.

IFN-γ stimulates the production of neopterin by macrophages and also increases the conversion of tryptophan to kynurenine through increased activity of IDO. We observed a decrease in KTR, though levels of neopterin did not change significantly after weight loss. This might indicate that mechanisms beyond IFN-γ activity contribute to the regulation of tryptophan to kynurenine conversion in patients with obesity. The metabolism of tryptophan to kynurenine is also catalyzed by TDO, which is induced by stress hormones, such as cortisol [11]. Patients with severe obesity and metabolic impairment have increased levels of cortisol [30]. Thus, a decrease in obesity-related cortisol and TDO activation might contribute to the observed reduction in KTR after weight loss.

Low levels of PLP are linked to conditions associated with inflammation, such as CVD and metabolic syndrome [31, 32], but the mechanisms by which PLP is decreased are not fully understood. Increased inflammation seems to be associated with higher cellular uptake and catabolism of vitamin B6 [33]. Enhanced demand of PLP as a cofactor might lead to functional deficiency [34]. Additionally, stress might cause PLP deficiency by activation of the PLP phosphatase due to increased levels of cortisol [35]. Thus, an increase in vitamin B6 status after bariatric surgery as seen in our study might reflect reduced inflammation. The increase was higher between three months and one year after surgery than between baseline and three months. The decrease in the ratios between 3-hydroxykynurenine:xanturenic acid, 3-hydroxykynurenine:3-hydroxyanthranilic acid and 3-hydroxykynurenine:kynurenic acid between three months and one year after surgery might be related to the increased PLP.

Circulating levels of the vitamin B3 complex nicotinamide and N^1^-methylnicotinamide decreased during the first three months after surgery, despite supplementation of vitamin B3. Vitamin B3 is obtained directly from the diet or synthesized from dietary tryptophan [36]. The observed decrease in vitamin B3 is possibly caused by restricted food uptake after bariatric surgery, as also reflected by lower levels of tryptophan.

At baseline the kynurenine metabolites 3-hydroxykynurenine and 3-hydroxyanthranilic acid were positively correlated with HbA1c. Circulating concentrations of both these tryptophan metabolites decreased significantly after weight loss. In addition, changes in 3-hydroxykynurenine one year after bariatric surgery were positively correlated to changes in HbA1c and the TG:HDL-ratio. The formation of 3-hydroxykynurenine is dependent on the enzyme kynurenine 3-monooxygenase (KMO), which converts kynurenine to 3-hydroxy-L-kynurenine. KMO is present in macrophages in adipose tissue and it has been shown that KMO activity is positively correlated to HbA1c levels [37]. Additionally, elevated circulating levels of 3-hydroxykynurenine were shown in diabetic individuals with retinopathy [38]. Patients who were operated using BPD-DS had higher incidence of T2D than the patients operated with LGS and at baseline these patients also had higher levels of 3-hydroxyanthranilic acid. 3-Hydroxyanthranilic acid is a downstream metabolite of 3-hydroxykynurenine, dependent on the enzyme kynureninase. Possibly, understanding the role of 3-hydroxykunurenine and 3-hydroxyanthranilic acid could help elucidate why some individuals with obesity have an increased risk of developing metabolic impairment compared to metabolically healthy individuals with obesity.

Anthranilic acid was the only kynurenine metabolite measured that increased after weight loss. A different regulation of anthranilic acid compared to other kynurenines measured in individuals with obesity has previously been described [29]. Conversion of kynurenine into anthranilic acid is catalysed by the PLP-dependent enzyme kynureninase [11], and anthranilic acid increases in response to pyridoxine supplementation [39]. The increase in circulating PLP after weight loss might explain some of the observed increase in anthranilic acid. Possibly, also a reduction in the KMO activity after weight loss may favour the formation of anthranilic acid instead of 3-hydroxykynurenine. Further studies on the role of anthranilic acid in inflammatory conditions are needed.

It should be noted that patients in this study received multi-vitamin supplements containing vitamin B2, B3 and B6 after surgery. This makes it difficult to evaluate the effect of reduced inflammation vs. supplements or impaired vitamin uptake on measured levels of vitamin B3 and B6. Although, supplementation of pyridoxine does not normalize the circulating levels of inflammatory markers [40], vitamin intake was adjusted for in our analysis. Additionally, the major changes in B-vitamin levels and reduction in inflammatory markers occurred between 3 months and one year after surgery, which might indicate that the increase in B vitamins were not mainly caused by increased intake through supplements, but rather the metabolic and inflammatory changes occurring during weight loss. Two different types of bariatric surgery were used in this study, LSG and BPD-DS. The type of bariatric surgery performed possibly plays a role regarding weight loss, metabolic improvement and decreased inflammation. Due to the small sample size the present study was not designed to evaluate differences in the surgical procedures.

## Conclusion

In summary, strong correlations between HbA1c and the kynurenine metabolites 3-hydroxykynurenine and 3-hydroxyanthranilic acid were observed in patients with obesity scheduled for bariatric surgery. Following surgery, vitamin B6 levels increased whereas KTR and CRP decreased, indicating down-regulated inflammation after profound weight loss. The role of kynurenine metabolites in the pathophysiologic pathways leading to metabolic impairment in individuals with obesity needs further evaluation.

## Supporting information

S1 Appendix Data file. (XLSX)
